# Antiestrogenic and Anti-Inflammatory Potential of *n*-Hexane Fraction of *Vitex negundo* Linn Leaf Extract: A Probable Mechanism for Blastocyst Implantation Failure in *Mus musculus*

**DOI:** 10.1155/2014/241946

**Published:** 2014-10-29

**Authors:** Mehul Jivrajani, Nirav Ravat, Sheetal Anandjiwala, Manish Nivsarkar

**Affiliations:** ^1^Department of Pharmacology and Toxicology, B. V. Patel Pharmaceutical Education and Research Development (PERD) Centre, Sarkhej-Gandhinagar Highway, Thaltej, Ahmedabad, Gujarat 380 054, India; ^2^Department of Natural Products, National Institute of Pharmaceutical Education and Research (NIPER), Ahmedabad, Gujarat 380054, India

## Abstract

The anti-implantation potential of different fractions of *Vitex negundo* Linn leaf extract was evaluated in female *Swiss Albino* mice. Animals from different groups were dosed orally either with 0.2% agar (vehicle) or with fractions of *V. negundo* leaf extract (*n*-hexane, chloroform, *n*-butanol, and remnant fractions) at 10:00 a.m., from day 1 to day 6 of pregnancy. The pregnant females from each group were sacrificed on different days of pregnancy (*n* = 6), and uterus was excised and used for estimation of lipid peroxidation and assay of superoxide dismutase activity as a marker for blastocyst implantation. Animals treated with *n*-hexane fraction showed altered level of superoxide anion radical and superoxide dismutase activity as compared to control animals. The probable mechanism by which this extract exhibits inhibition of blastocyst implantation is through the anti-inflammatory and antiestrogenic potential.

## 1. Introduction

The process of implantation is required for nearly all viviparous births and is a very critical event in reproductive physiology. Many biochemical, biophysical, and hormonal changes occur prior to this event [1]. One of the crucial aspects in this whole reproduction process is “endometrial receptivity” which is necessary for blastocyst implantation [2]. Endometrial receptivity is for a self-limited period, in which the endometrial epithelium acquires a functional and transient ovarian steroid-dependent status that allows blastocyst adhesion [3]. This receptive endometrial state is defined as the “window of implantation.”

The process of implantation is modulated by various “proinflammatory” mediators along with the ovarian hormones. Studies have shown that various inflammatory mediators, such as tumor necrosis factor-*α* (TNF-*α*), nitric oxide (NO), superoxide anion radical, interleukin-1 (IL-1), leukemia inhibitory factor (LIF), and colony-stimulating factor (CSF-1), are involved in the process of implantation [4–7]. Prostaglandins (PGs) are also implicated as important mediators for increased endometrial vascular permeability during implantation [8, 9]. This phenomenon is evident from delayed implantation or implantation failure by COX1 and COX2 inhibitors in dose dependant manner in mice [9]. Hence, it is clear that there exists a possibility that any compound showing good anti-inflammatory activity may show significant anti-implantation activity.

It is also reported that the recruitment of these proinflammatory mediators at the site of implantation is modulated by the ovarian steroid hormones (estrogen and progesterone). Proinflammatory molecules which are useful during implantation are secreted by leucocytes in response to changes in these hormonal levels [4] especially estrogen. Thus, it can be hypothesized that any compound showing good antiestrogenic activity may also downregulate these proinflammatory molecules and inhibit blastocyst implantation.

Our previous studies on* Vitex negundo* leaf extract showed potential anti-implantation activity [10]. The leaf extract was further fractionated to evaluate this concept. Different fractions of* Vitex negundo* Linn leaf extract were screened for anti-implantation activity.* V. negundo* Linn is a large aromatic shrub from Verbenaceae family and is found throughout India especially in the warmer zones [11]. It has been claimed to possess many medicinal properties. Almost all of its parts are used in Indian systems of medicine to treat various diseases. Leaves and seeds of* V. negundo* were used traditionally for treatment of rheumatism and inflammatory disorder [12].* V. negundo* has also been studied extensively for its anti-inflammatory and analgesic activities [13], antioxidant properties [14], anticataract activity [15], and free radical scavenging activity [16]. Furthermore, seeds and leaves of* V. negundo* also show antifertility [17] and anti-implantation [10] activities.

The objective of the present work was to investigate and compare the anti-implantation potential of different fractions of* Vitex negundo* Linn leaf extract and to identify the active fraction responsible for the anti-implantation activity.

## 2. Materials and Methods

### 2.1. Animals

Mature, female mice (*Swiss Albino* strain,* Mus musculus*, 2-3 months old) housed in temperature-controlled (27 ± 1°C) room at light : dark regimen of 14 : 10 hours were used for the study. The experimental protocol was approved by the Institutional Animal House Ethics Committee (IAEC), constituted by the Ministry of Social Justice and Empowerment, Government of India. Only those female mice that showed a regular 4-5 days of estrous cycle were used in the study. Vaginal smears were examined daily according to the guidelines provided by Caligioni [18]. Females that showed a proestrus smear (day 0) were mated with a male of proven fertility on the same evening. Presence of a vaginal plug (next day morning) confirmed mating and was designated as day 1 of pregnancy. The pregnant females were sacrificed on days 1, 2, 3, 4, 5 (4:40 a.m.), 5 (10:00 am), and 6 of pregnancy. The uterus was excised from each animal, cleaned from adhering fat, and washed with normal saline. Appropriately weighed uterus was used for estimation of lipid peroxidation and assay of superoxide dismutase (SOD) activity.

### 2.2. Chemicals

Pyrogallol (pyrogallic acid; 1,2,3-trihydroxybenzene), SDS (sodium dodecyl sulphate), Chicago blue 6B dye, and TBA (2-thiobarbituric acid) were purchased from HiMedia Laboratories, Mumbai, India. Triton X 100 and ethinyl estradiol were obtained from Sigma Aldrich (Milwaukee, WI, USA). *β*-Sitosterol was purchased from Natural Remedies Pvt. Ltd., Bangalore, India, and lupeol was a gift sample from S. C. Pal College of Pharmacy, Nasik, India. Agar was purchased from Merck, Mumbai, India. All the solvents used were of analytical grade and were obtained from Merck, Mumbai, India. Deionized water used in the experiment was prepared in-house using a water purifier system (Millipore Elix, Germany).

### 2.3. Bioactivity Guided Fractionation of Plant* Vitex negundo* Linn

Fresh green leaves of* V. negundo* were collected from the botanical garden of B. V. Patel Pharmaceutical Education and Research Development (PERD) Centre, Ahmedabad, and were authenticated. A voucher specimen was preserved. Leaves were washed, shade-dried, and powdered. Accurately weighed 500 g of the powdered drug was extracted with methanol (1000 mL × 3) under reflux on a water bath. The extract was filtered through Whatman filter paper number 1. Extract was pooled and concentrated under vacuum to dryness to obtain crude methanolic extract (~90 g). This extract was then resuspended in water and partitioned successively in a separating funnel using organic solvents of increasing polarity, namely,* n*-hexane (250 mL × 3), chloroform (250 mL × 3), and* n*-butanol (250 mL × 3). Solvents were evaporated under vacuum to obtain fraction of* n*-hexane (9.429 g), chloroform (11.625 g),* n*-butanol (45.375 g), and remnant (22.724 g), respectively. Extracts were stored in the refrigerator at 4°C until further use. For pharmacological studies, the fractions were suspended in a 0.2% aqueous agar solution. The doses employed were expressed as milligram of the dried extract per kilogram body weight.

### 2.4. TLC Fingerprint Profile

*n*-Hexane fraction (100 mg) of* V. negundo* leaves was dissolved in 40 mL of* n*-hexane and the volume was made up to 50 mL in a volumetric flask. This fraction was used for the TLC (thin layer chromatography) fingerprinting profile. TLC plates consisted of 10 × 10 cm, precoated with silica gel 60 F_254_ TLC plates (E. Merck) (0.2 mm thickness) with aluminum sheet support. The spotting device was a CAMAG Linomat V Automatic Sample Spotter (Camag Muttenz, Switzerland); the syringe was 100 *µ*L (Hamilton). The developing chamber was a CAMAG glass twin trough chamber (20 × 10 cm), densitometer a Camag TLC Scanner 3 linked to winCATS software. The experimental conditions were kept constant where temperature was 25 ± 2°C and relative humidity was 40%. TLC fingerprint was developed by applying 25 *µ*L of* n*-hexane fraction (100 mg/50 mL) in duplicate along with standards, lupeol, and *β*-sitosterol with band distance of 12 mm and band size of 8 mm. Plate was developed in a solvent system of toluene : methanol (9.7 : 0.3), dried and observed under UV 254 nm and UV 366 nm. The plate was derivatized with anisaldehyde-sulfuric acid reagent followed by heating at 100°C until the colored band appeared. The *R*_*F*_ value and color of the resolved bands were noted.

### 2.5. Simultaneous Quantification of Lupeol and *β*-Sitosterol in the Sample

*n*-Hexane fraction (100 mg) of* V. negundo* leaves was dissolved in 40 mL of* n*-hexane and the volume was made up to 50 mL in a volumetric flask. This fraction was used for the quantification of lupeol and *β*-sitosterol. Fifteen microliters of this dilution was applied in triplicate on a TLC plate along with standards, lupeol, and *β*-sitosterol. The plate was developed as mentioned above and scanned at 525 nm. The peak areas were recorded and the amount of lupeol and *β*-sitosterol was calculated using the respective calibration curves.

### 2.6. Treatment of Animals

Animals were divided into five different groups, groups A to E. Animals from groups A, B, C, D, and E were dosed orally at 10:00 a.m. each day from day 1 to day 6 of pregnancy with 0.2% agar (control),* n*-hexane fraction (52 mg/kg body weight), chloroform fraction (64 mg/kg body weight),* n*-butanol fraction (252 mg/kg body weight), and remnant fraction (126 mg/kg body weight). The pregnant females from each group were sacrificed on days 1, 2, 3, 4, 5 (4:40 a.m.), 5 (10:00 a.m.), and 6 of pregnancy. The uterus was excised, cleaned from adhering fat, washed with normal saline, weighed, and then used for estimation of lipid peroxidation and assay of superoxide dismutase (SOD) activity. A second set of animals (*n* = 6) from each group were injected intravenously 0.1 mL of 1% Chicago blue 6B dye through tail vein on day 6 of pregnancy. All the animals were sacrificed and the uterus was exposed to count the number of sites of implantation.

### 2.7. Estimation of Lipid Peroxidation

Uterine tissue was taken in 5 mL of Hank's balanced salt solution (HBSS, pH 7.4) and homogenized at 5000 rpm, using a Polytron homogenizer (3 cycles of 30 sec each; Kinematica, Switzerland). The homogenate was then centrifuged at 3500 rpm for 10 minutes (min). The pellet was resuspended in 0.1 mL of HBSS and used for estimation of lipid peroxidation. Lipid peroxidation was measured in terms of malondialdehyde (MDA) : thiobarbituric acid (TBA) reaction as reported by Ohkawa et al. [19]. The reaction mixture contained 0.1 mL of tissue homogenate (as described above), 0.2 mL of 8.1% sodium dodecyl sulphate, 1.5 mL of 20% acetic acid (pH adjusted to 3.5 with 1 M NaOH), and 1.5 mL of 0.8% aqueous solution of TBA. The reaction mixture was made to a volume of 4 mL with the addition of 0.7 mL of double distilled water and heated at 95°C for 1 hour in a water bath. After heating, 1 mL of double distilled water and 5 mL of a mixture of* n*-butanol and pyridine (15 : 1 v/v) were added and the mixture was shaken vigorously on a vortex mixer for 5 min. This mixture was then centrifuged at 3000 rpm for 7 min. After centrifugation the upper organic layer was separated and the amount of MDA formed in this layer was measured at 532 nm using an ultra violet/visible spectrophotometer (Systronics, India). Appropriate controls were used at different steps during this estimation (extinction coefficient of MDA is 1.45 × 10^−5^/min/cm).

### 2.8. Assay of Superoxide Dismutase Activity

The uterine tissue was taken in 4 mL of chilled Tris buffer (50 mM pH 8.2) and was homogenized at 13,000 rpm (3 cycles of 30 sec each), using a Polytron homogenizer. The homogenate was treated with 1 mL of 0.1% Triton X 100 (v/v) for 20 min at 4°C. Homogenate was then centrifuged at 10,000 rpm at 4°C for 30 min using Sorvall, legend Micro21R centrifuge. The supernatant was used for the assay of superoxide dismutase (SOD) activity by the method as reported by S. Marklund and G. Marklund [20]. All calculations were made as per gram fresh weight.

### 2.9. Estimation of Antiestrogenic Property

Healthy virgin female mice,* Swiss Albino*, were divided into four groups (*n* = 6). One group received* n*-hexane fraction of* V. negundo* leaf extract alone (52 mg/kg body weight) (designated as a control), another group received ethinyl estradiol alone (100 *µ*g), the third group received both extract and ethinyl estradiol, and the fourth group consisted of normal animals (designated as untreated). Antiestrogenic activity was determined after daily administration of extract and subcutaneous injection of ethinyl estradiol for 7 days. Uterine weight at the end of the experiment was used as a parameter for antiestrogenic property [21].

### 2.10. Statistical Analysis

All data have been represented as mean ± SEM. Data were analyzed using paired* t*-tests within groups and *P* < 0.05 was considered significant. Linear correlation was established between the LPO and SOD values on day 5 (4.40 a.m.) and the correlation coefficient was calculated.

## 3. Results

### 3.1. TLC Fingerprint Profile and Simultaneous Quantification of Lupeol and *β*-Sitosterol

**Figure 1:**
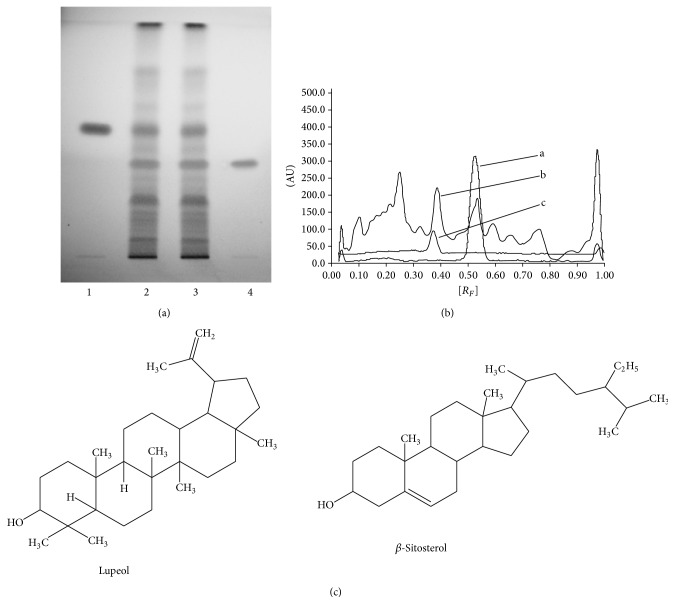
(a) TLC fingerprint profile of* n*-hexane fraction of* Vitex negundo* leaves. (1) Lupeol standard; (2) and (3)* n*-hexane fraction; (4) *β*-sitosterol standard. (b) TLC densitometric chromatogram of* n*-hexane fraction of* Vitex negundo* at 525 nm. a: Lupeol standard; b:* n*-hexane fraction; c: *β*-sitosterol. (c) Structure of lupeol and *β*-sitosterol.

Figure 1 shows the TLC fingerprint profile of the* n*-hexane fraction. The TLC showed presence of lupeol and *β*-sitosterol along with some other compounds (unknown) after derivatization (Figure 1(a)). The *R*_*F*_ value of lupeol and *β*-sitosterol was 0.53 and 0.35, respectively. Further, lupeol and *β*-sitosterol were quantified from the* n*-hexane fraction. The TLC plate was scanned at 525 nm to obtain the chromatogram of* n*-hexane fraction of* V. negundo* (Figure 1(b)). The linearity ranges for lupeol and *β*-sitosterol were found to be 150–900 and 80–480 ng/spot, with correlation coefficients (*r* values) of 0.999 and 0.991. The content of lupeol (Figure 1(c)) and *β*-sitosterol (Figure 1(c)) quantified using the TLC densitometric method was 2.39% w/w and 1.41% w/w, respectively. This amount is quite adequate to screen this fraction for the anti-implantation activity.

### 3.2. Anti-Implantation Activity of Solvent Fractions of* Vitex negundo* Leaf Extract

**Figure 2:**
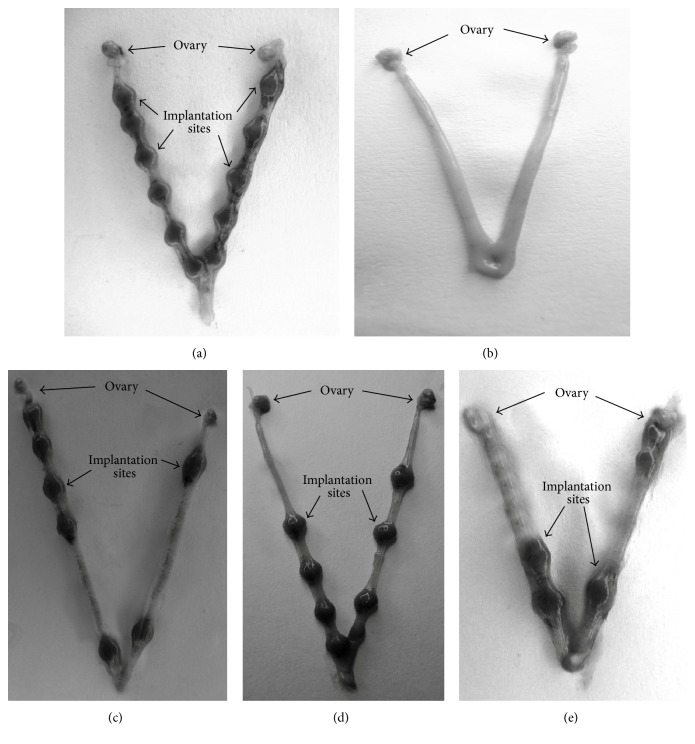
Uterus of (a) control mice showing blastocyst implantation sites; (b)* n*-hexane fraction of* Vitex negundo* Linn leaf extract treated mice, not showing any implantation site; (c) chloroform fraction; (d)* n*-butanol fraction; and (e) remnant fraction of* Vitex negundo* Linn leaf extract treated mice shows partial inhibition of implantation.

Animals treated with* n*-butanol, chloroform, and remnant fraction of* Vitex negundo* leaf extract for the first six days of pregnancy showed partial inhibition in blastocyst implantation, whereas animals treated with* n*-hexane fraction of* V. negundo* leaf extract showed complete inhibition in blastocyst implantation (Table 1) (Figure 2).

**Figure 3:**
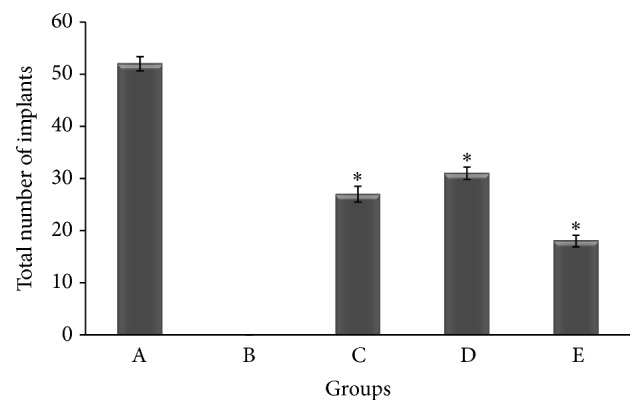
Graph showing total number of implants in uterus of (A) control mice, (B)* n*-hexane fraction, (C) chloroform fraction, (D)* n*-butanol fraction, and (E) remnant fraction of* Vitex negundo* Linn leaf extract treated mice, respectively. ^*^ *P* < 0.05 was considered significant when compared to group of control animals.

The uterus of control animals showed implantation sites (Figure 2(a)). No implantation sites were observed in the uterus from treatment group animals (*n*-hexane fraction of* V. negundo* leaf extract) (Figure 2(b)). Similarly, Figure 3 depicts graph showing total number of implants in uterus of control mice and mice treated with* n*-hexane fraction, chloroform fraction,* n*-butanol fraction, and remnant fraction of* Vitex negundo* Linn leaf extract treated mice, respectively. Even though animals treated with* n*-butanol, chloroform, and remnant fraction of* Vitex negundo* leaf extract showed partial inhibition in blastocyst implantation, inhibition is statistically significant when compared to control animals. No implantation site has been observed in mice treated with* n*-hexane fraction of* V. negundo* leaf extract (Figure 3).

### 3.3. Lipid Peroxidation and Superoxide Dismutase Enzyme Activity

**Figure 4:**
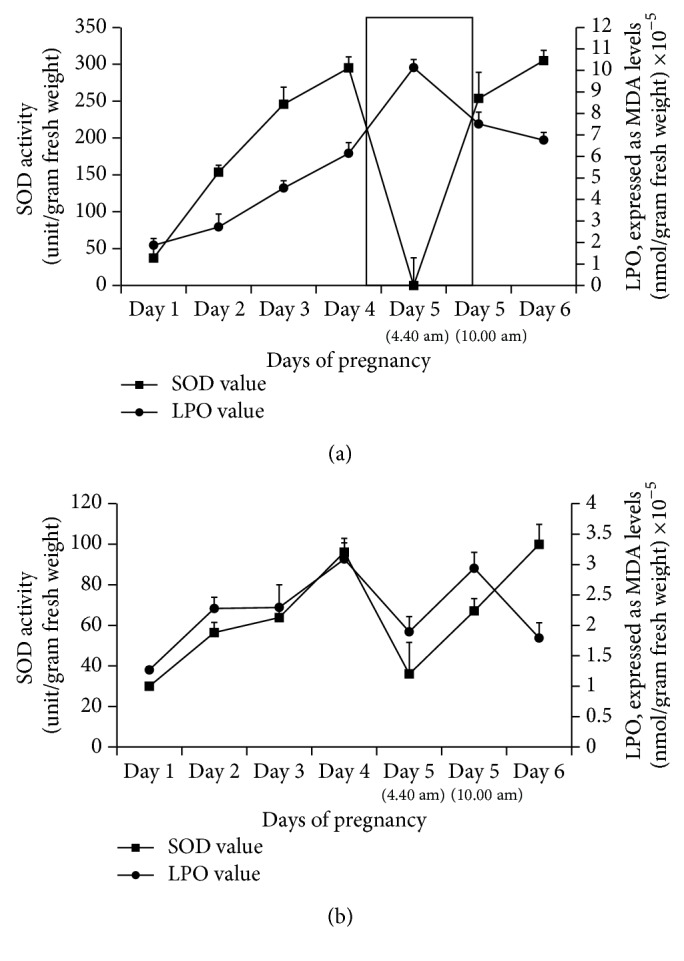
(a) Superoxide dismutase (SOD) activity and lipid peroxidation (LPO; MDA levels) on different days of pregnancy (days 1–6) in uterus of control animals (*n* = 6) (*r* = −0.902). Selected area shows “window of implantation.” (b) Superoxide dismutase (SOD) activity and lipid peroxidation (LPO; MDA levels) on different days of pregnancy (days 1–6) in uterus of* n*-hexane fraction of* Vitex negundo* Linn leaf extract treated animals (*n* = 6) (*r* = 0.157).

SOD (superoxide dismutase) and superoxide anion radical levels (measured as MDA levels) were measured in the uterus of control and treated animals, from days 1 to 6 of pregnancy. SOD and superoxide anion radical were used as a marker for blastocyst implantation [10, 22, 23]. Control animals showed a sharp decrease in SOD levels and a sharp increase in superoxide anion radical at the time of implantation (day 5, 4:40 a.m., *P* < 0.05) when compared to days 4 and 5 (10:00 a.m.) of pregnancy. A negative correlation was found (*r* = −0.902) between the levels of superoxide anion radical and SOD in the uterus at the time of blastocyst implantation (Figure 4(a)). Animals treated with chloroform,* n*-butanol, and remnant fraction of* V. negundo* leaf extract showed almost similar pattern as control animals (data not shown).

However, in the* n*-hexane fraction treated animals, levels of superoxide anion radical and SOD were altered during the blastocyst implantation. A sharp decrease in superoxide anion radical and increase in SOD activity were observed (*r* = 0.1570) when compared to days 4 and 5 (10:00 a.m.) of pregnancy (Figure 4(b)). This difference in superoxide anion radical and SOD activity between control and* n*-hexane treated animals is statistically significant (*P* < 0.05).

### 3.4. Antiestrogenic Potential

**Figure 5:**
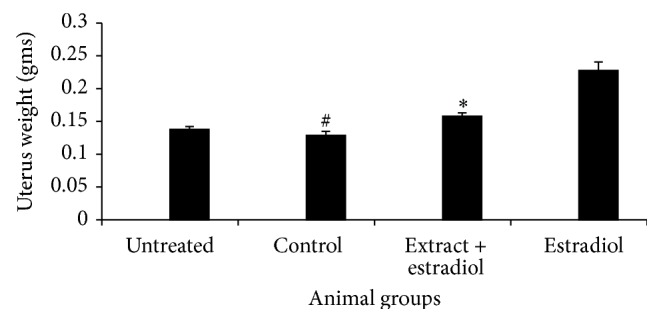
Uterine weight in untreated, control (extract alone), ethinyl estradiol plus* n*-hexane fraction of* Vitex negundo* Linn leaf extract and ethinyl estradiol alone treated animals, respectively (*n* = 6). ^#, ∗^ *P* < 0.05 was considered significant when compared to ethinyl estradiol alone treated animals.

Antiestrogenic potential of the* n*-hexane fraction was evaluated. Animals treated with both the extract and ethinyl estradiol exhibited decrease in uterine weight as compared to animal treated with ethinyl estradiol alone (*P* < 0.05) (Figure 5), which suggests the antiestrogenic property of the* n*-hexane fraction.

## 4. Discussion

TLC fingerprint profile of* n*-hexane fraction clearly showed the presence of lupeol and *β*-sitosterol. Both lupeol and *β*-sitosterol are previously reported for anti-inflammatory and antioxidant activity [24, 25].

Animal experiment results clearly indicate that implantation failure cannot be due to the interference with tubal transport of the fertilized egg. This phenomenon can be attributed to the hostile environment of the endometrium because of the* n*-hexane fraction. The preimplantation and peri-implantation periods of the embryo and the endometrium include a number of biochemical, biophysical, and molecular changes in the endometrial and blastocyst membrane. The successful completion of these events involves a complex series of synchronized changes in the blastocyst and the endometrium. Interaction between the blastocyst and the endometrium is mediated by number of signals generated by molecules such as cytokines, growth factors, free radicals, and adhesion molecules which are produced and/or secreted by the endometrium and blastocyst [5, 6].

Membrane fluidity, a prerequisite for implantation, is achieved by all the above mediators. The arrival of the zygote in the endometrium is not sufficient to ensure implantation; hormone-dependent changes and the increase in membrane fluidity, also called “receptive endometrium,” are required for successful blastocyst implantation. The establishment of the receptive endometrium to support embryo development and implantation is primarily coordinated by ovarian hormones, progesterone, and estrogen that modulate uterine events. A fine balance of estrogen and progesterone exists in the uterine milieu and has critical role in blastocyst implantation. For instance, implantation requires a preovulatory increase in the secretion of estrogen, which stimulates the proliferation and differentiation of uterine epithelial cells. In the same way, continued production of progesterone by the corpus luteum stimulates the proliferation and differentiation of stromal cells. Even though, both estrogen and progesterone are required for blastocyst implantation, estrogen is a crucial determinant of the window of implantation in mice. McCormack and Greenwald [26] have demonstrated that, in mouse, estrogen plasma level starts to increase from 9th hour, peak at 11th hour on day 4 of pregnancy, and persist for 24 hours [26]. In mouse, blastocyst implantation occurs early morning on day 5 which suggests the critical role of estrogen in blastocyst implantation. It has been previously reported that implantation can be induced in the ovariectomized pregnant mice by single injection of estrogen, which confirms the vital role of estrogen in implantation [27]. The period from 10th to 12th hour on day 4 (time of estrogen peak) appears to be crucial because ovariectomy after these hours does not inhibit implantation [26]. The superoxide anion radical surge at the time of implantation has been implicated for induction of endometrial membrane fluidity (“receptive endometrium”) [1]. An estrogen surge at the time of implantation has been shown to be responsible for a decrease in SOD levels and an increase in superoxide anion radical levels [28].

Animals treated with* n*-hexane fraction of* V. negundo* showed very low superoxide anion radical level resulting in “nonreceptive endometrium,” which failed to accept blastocyst and finally led to implantation failure. Failure in blastocyst implantation may be attributed to antiestrogenic and anti-inflammatory properties of* n*-hexane fraction of* V. negundo*. Antiestrogenic property of* n*-hexane fraction of* V. negundo* was confirmed by decrease in uterine weight upon administration of the extract. Additionally, the* n*-hexane fraction of* V. negundo* is rich in triterpenoids like lupeol and *β*-sitosterol, which are good anti-inflammatory agents [29]. Presence of these agents may downregulate the various mediators such as cyclooxygenase, various cytokines, nitric oxide, and superoxide anion radical. However, as* n*-hexane fraction shows very good antiestrogenic activity, it may also downregulate these mediators. Hence,* n*-hexane fraction of* V. negundo* modulates two different yet interrelated pathways, inflammatory cascade and estrogen signaling, resulting in alteration in the physiology of the endometrium making it “nonreceptive” which further results in the failure of blastocyst implantation.

## 5. Conclusions

*n*-Hexane fraction of* Vitex negundo* leaf extract exhibits potent anti-implantation activity. The data suggests that high level of superoxide anion radical, which is prerequisite for the endometrial membrane fluidity, decreases upon administration of the extract. Moreover, anti-implantation activity presented by* n*-hexane fraction is also due to its antiestrogenic potential. Inhibition of estrogen is responsible for an increase in SOD level and decrease in superoxide anion radical level. Further, it also downregulates several inflammatory mediators like cyclooxygenase, various cytokines, nitric oxide, and so forth. Hence, the probable mechanism by which this extract exhibits inhibition of blastocyst implantation is through anti-inflammatory activity and antiestrogenic potential.

## Figures and Tables

**Table 1:** Blastocyst implantation sites in control and treated animals.

Groups	Number of pregnant females	Number of implants/number of animals
A	6	52/6
B	6	0/6
C	6	27/6
D	6	31/6
E	6	18/6
